# Vision and 2D LiDAR Fusion-Based Navigation Line Extraction for Autonomous Agricultural Robots in Dense Pomegranate Orchards

**DOI:** 10.3390/s25175432

**Published:** 2025-09-02

**Authors:** Zhikang Shi, Ziwen Bai, Kechuan Yi, Baijing Qiu, Xiaoya Dong, Qingqing Wang, Chunxia Jiang, Xinwei Zhang, Xin Huang

**Affiliations:** 1 College of Intelligent Manufacturing, Anhui Science and Technology University, Chuzhou 239000, China; shizk1212@163.com (Z.S.); baizw@ahstu.edu.cn (Z.B.); yikc@ahstu.edu.cn (K.Y.); qqwang@ahstu.edu.cn (Q.W.); jiangcx@ahstu.edu.cn (C.J.); zhangxw@ahstu.edu.cn (X.Z.); 2 Key Laboratory of Plant Protection Engineering, Ministry of Agriculture and Rural Affairs, Jiangsu University, Zhenjiang 212013, China; qbj@ujs.edu.cn (B.Q.); dongxiaoya@ujs.edu.cn (X.D.); 3 Key Laboratory of Modern Agricultural Equipment and Technology, Ministry of Education, Jiangsu University, Zhenjiang 212013, China

**Keywords:** orchard navigation, sensor fusion, object detection, DBSCAN algorithm, RANSAC algorithm, agricultural robots

## Abstract

To address the insufficient accuracy of traditional single-sensor navigation methods in dense planting environments of pomegranate orchards, this paper proposes a vision and LiDAR fusion-based navigation line extraction method for orchard environments. The proposed method integrates a YOLOv8-ResCBAM trunk detection model, a reverse ray projection fusion algorithm, and geometric constraint-based navigation line fitting techniques. The object detection model enables high-precision real-time detection of pomegranate tree trunks. A reverse ray projection algorithm is proposed to convert pixel coordinates from visual detection into three-dimensional rays and compute their intersections with LiDAR scanning planes, achieving effective association between visual and LiDAR data. Finally, geometric constraints are introduced to improve the RANSAC algorithm for navigation line fitting, combined with Kalman filtering techniques to reduce navigation line fluctuations. Field experiments demonstrate that the proposed fusion-based navigation method improves navigation accuracy over single-sensor methods and semantic-segmentation methods, reducing the average lateral error to 5.2 cm, yielding an average lateral error RMS of 6.6 cm, and achieving a navigation success rate of 95.4%. These results validate the effectiveness of the vision and 2D LiDAR fusion-based approach in complex orchard environments and provide a viable route toward autonomous navigation for orchard robots.

## 1. Introduction

As an important economic crop, pomegranate cultivation has developed rapidly in many regions [1,2]. However, the level of mechanization in orchard operations significantly lags behind other agricultural sectors. Particularly in densely planted pomegranate orchards, traditional manual operations are not only labor-intensive but also exhibit low efficiency, severely constraining the rapid development of the pomegranate industry [3].

**Figure 1:**
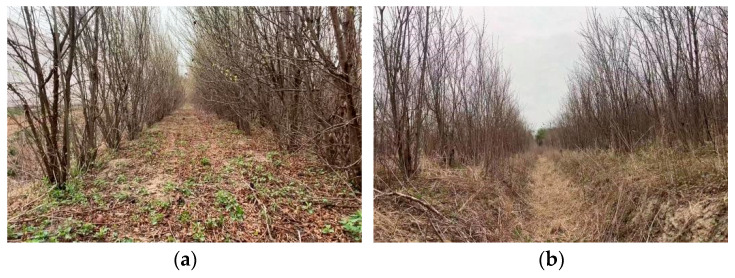
Pomegranate orchard environment: (**a**) Regular planting pattern; (**b**) Slope covering planting pattern.

Compared with other orchards, pomegranate orchards exhibit distinct environmental characteristics and operational challenges. At the young-tree stage, trunk diameters are only 5–15 cm, and the planting density is high, with a row spacing of 2–3 m and an in-row spacing of 1–2 m, as shown in Figure 1. Beyond the spatial layout, ambient conditions directly affect both experiments and algorithm performance: fluctuations in temperature and humidity broaden the image luminance dynamic range; strong direct sunlight and dappled canopy shadows cause unstable image contrast and textures, increasing the difficulty of trunk detection; the ground surface contains both bare soil and weeds, and the soil is predominantly sandy loam—under dry conditions dust is raised, whereas under wet conditions water-surface glare and wheel slip further degrade the measurement quality of the camera and LiDAR. These climate, soil, and ground-surface factors, compounded with the high-density planting structure, impose more demanding operating conditions than typical orchards, making autonomous navigation more challenging in object perception, robust fitting, and path tracking [4,5].

In the development of autonomous navigation technology for orchards, traditional orchard navigation methods primarily rely on Global Navigation Satellite Systems (GNSS). However, in dense canopy-occluded orchard environments, GNSS signals are susceptible to interference or loss, resulting in significantly degraded positioning accuracy [6]. To overcome this limitation, researchers have developed sensor-based active positioning and navigation technologies, among which Light Detection and Ranging (LiDAR) and machine vision technologies have attracted considerable attention due to their robustness in complex environments [7].

In the field of machine vision navigation, Liu et al. [8] developed a single-stage navigation path extraction network (NPENet) that directly predicts road centerlines through an end-to-end approach, simplifying traditional multi-stage processing workflows. Cao et al. [9] implemented grape trunk detection based on an improved YOLOv8-Trunk network and fitted navigation lines using the least squares method. Yang et al. [10] proposed a visual navigation path extraction method based on neural networks and pixel scanning, improving the segmentation performance for orchard road condition information and background environments. Silva et al. [11] presented a deep learning crop row detection algorithm capable of achieving robust visual navigation under different field conditions. Gai et al. [12] developed a depth camera-based crop row detection system particularly suitable for robot navigation under tall crop canopies such as corn and sorghum, addressing GPS signal occlusion issues. Winterhalter et al. [13] proposed a method using pattern Hough transform to detect small plants, improving the accuracy of crop row detection in early growth stages. Gai et al. [14] introduced a color and depth image fusion method that enhanced crop detection accuracy under high weed density conditions. However, these single vision-based methods experience significant performance degradation under adverse lighting conditions and struggle to directly obtain precise distance information, limiting their application in precision navigation [15].

Jiang et al. [16] developed a 2D LiDAR-based orchard spraying robot navigation system that achieved trunk detection and path planning through DBSCAN clustering and RANSAC algorithms. Li et al. [17] proposed a 3D LiDAR-based autonomous navigation method for orchard mobile robots, optimizing point cloud processing efficiency through octree data structures. Abanay et al. [18] developed a ROS-based 2D LiDAR navigation system for strawberry greenhouse environments. Liu et al. [19] presented a LiDAR-based navigation system for standardized apple trees, but it primarily relied on regularly arranged tree configurations. Malavazi et al. [20] developed an autonomous agricultural robot navigation algorithm based on 2D LiDAR, achieving crop row line extraction through an improved PEARL method that enables robust navigation without prior crop information. Jiang et al. [21] proposed a 3D LiDAR SLAM-based orchard spraying robot navigation system, combining NDT and ICP point cloud registration algorithms to improve positioning accuracy. Jiang et al. [22] developed a 3D LiDAR SLAM-based autonomous navigation system for stacked cage farming, achieving reliable robot localization and mapping. Firkat et al. [23] proposed FGSeg, a LiDAR-based field ground segmentation algorithm for agricultural robots, achieving high-precision ground detection and obstacle recognition through seed ground point extraction. However, these single LiDAR-based methods still face challenges of point cloud processing efficiency and high computational resource consumption in complex agricultural environments, with detection accuracy significantly declining when trunk diameters are small, limiting their widespread application in low-cost agricultural robots [24].

To overcome the limitations of single sensors, multi-sensor fusion technology has gradually become a research hotspot in the field of orchard navigation. Jiang et al. [25] employed thermal cameras and LiDAR as sensors, utilizing YOLACT (You Only Look At CoefficienTs) deep learning for navigation, object detection, and image segmentation, integrating accurate distance data from LiDAR to achieve real-time navigation based on vehicle position. Ban et al. [26] proposed a camera-LiDAR-IMU fusion-based navigation line extraction method for corn fields, achieving precise agricultural robot navigation through feature-level fusion. Kang et al. [27] presented a high-resolution LiDAR and camera fusion method for fruit localization, achieving fusion by projecting point clouds onto image planes. Han et al. [28] developed a LiDAR and vision-based obstacle avoidance and navigation system, using calibration parameters to transform both data types into the same coordinate system. Shalal et al. [29] developed an orchard mapping and mobile robot localization system based on onboard camera and laser scanner data fusion, achieving tree detection and precise robot positioning through geometric transformation registration of laser point clouds with visual images. Xue et al. [30] proposed a trunk detection method based on LiDAR and visual data fusion, employing evidence theory for multi-sensor information fusion and converting laser point clouds and visual images to a unified coordinate system through calibration parameters, improving trunk detection robustness. Yu et al. [31] noted in their review of agricultural 3D reconstruction technology that multi-sensor fusion technology combines LiDAR’s precise distance information with cameras’ rich texture information, providing more comprehensive environmental perception capabilities for precision agriculture. Ji et al. [32] proposed a farmland obstacle detection and recognition method based on fused point cloud data, achieving reliable obstacle detection in complex agricultural environments through multi-sensor data fusion. However, these multi-sensor fusion methods still face challenges in data synchronization, sensor calibration, and real-time processing, requiring further algorithm optimization to reduce computational complexity and improve system practicality [33]. Currently, specialized research on autonomous navigation for pomegranate orchards is relatively limited, with most studies focusing on mature orchard environments such as apple and citrus orchards [34], showing limited adaptability for dense planting scenarios.

In current agricultural robot navigation, many studies adopt conventional algorithm-based navigation line extraction methods, such as RANSAC-based navigation line fitting, simple fusion of sensor data, and SLAM-based global path planning. However, these existing methods often fail to cope effectively with densely planted orchards characterized by complex occlusions and diverse scene changes, especially when tree trunks are thin and the spacing between trunks is narrow. They are susceptible to environmental disturbances, occlusions, and illumination variations, leading to insufficient navigation accuracy and poor adaptability under complex conditions, and they cannot realize real-time orchard autonomous navigation without a prior map. To address these issues, this paper proposes a fusion-based navigation line extraction scheme that combines a YOLOv8-ResCBAM trunk detection model with 2D LiDAR data, aiming to tackle the real-time autonomous navigation challenges in densely planted orchards that current techniques struggle with and to improve navigation accuracy and stability. This research employs a YOLOv8-ResCBAM trunk detection network model based on improved YOLOv8 [35], enhancing target trunk detection accuracy in dense environments through the introduction of residual connections and dual attention mechanisms. A reverse ray projection fusion algorithm is proposed that differs from traditional point cloud-to-image plane projection methods [36], projecting visual detection information into three-dimensional space and computing intersections with LiDAR scanning planes to achieve precise data association. A navigation line fitting algorithm based on geometric constraints and directional confidence is constructed, improving RANSAC algorithm fitting accuracy and stability through analysis of inter-tree geometric relationships and construction of angle histograms. The technical contributions of this research provide references for solving autonomous navigation problems in densely planted orchard environments and hold significant practical value for advancing orchard mechanization and intelligent development.

## 2. Materials and Methods

### 2.1. Experimental Platform

**Figure 2:**
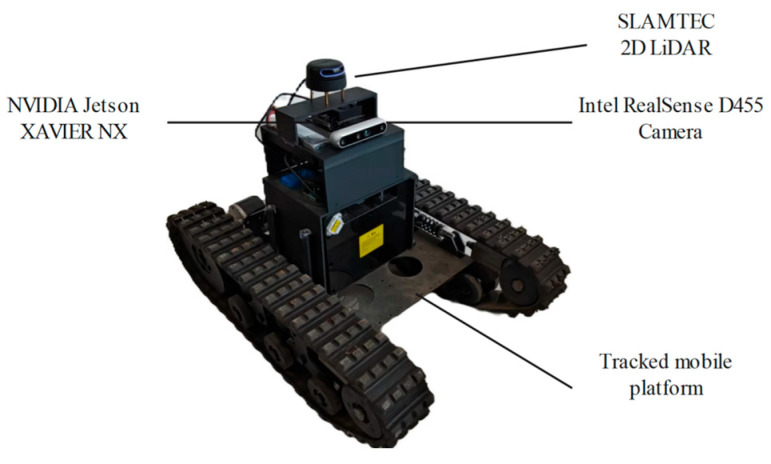
Experimental platform.

To validate the effectiveness of the proposed navigation system in pomegranate orchard environments, this study constructed an autonomous mobile platform as the experimental setup, as shown in Figure 2. The platform integrates an Intel RealSense D455 camera (Intel Corporation, Santa Clara, CA, USA), a SLAMTEC 2D LiDAR (Slamtec Co., Ltd., Shanghai, China), and an NVIDIA Jetson Xavier NX edge-computing module (NVIDIA Corporation, Santa Clara, CA, USA), and other equipment. The parameters of each component in the experimental platform are presented in Table 1.

The communication architecture of the entire system is based on the ROS Topics mechanism. Sensor data (camera images and LiDAR point clouds) are published as corresponding topics through their respective ROS drivers. The trunk detection module subscribes to the camera image topic and publishes detection results (bounding box center coordinates and confidence scores) as new ROS topics. The navigation line fitting module simultaneously subscribes to both the visual detection results topic and the LiDAR point cloud topic, and after fusion processing, publishes the fitted navigation line parameters to the path tracking control module. The path tracking control module receives the navigation line information, calculates the required linear and angular velocity control commands, and publishes them to the chassis motion controller through ROS Topics, thereby achieving autonomous navigation control of the mobile platform.

### 2.2. Monocular Camera and 2D LiDAR Data Fusion

In the complex autonomous navigation process of pomegranate orchards, accurate fruit tree positioning is the key to achieving orchard navigation. Sensor calibration aims to realize data fusion between vision and LiDAR to obtain accurate orchard information. This study conducted camera intrinsic parameter calibration, camera-LiDAR extrinsic parameter calibration, and joint calibration of multiple sensors to the chassis coordinate system, ensuring accurate alignment of different sensor data within a unified coordinate system [37].

#### 2.2.1. Coordinate System Definition and Establishment

**Figure 3:**
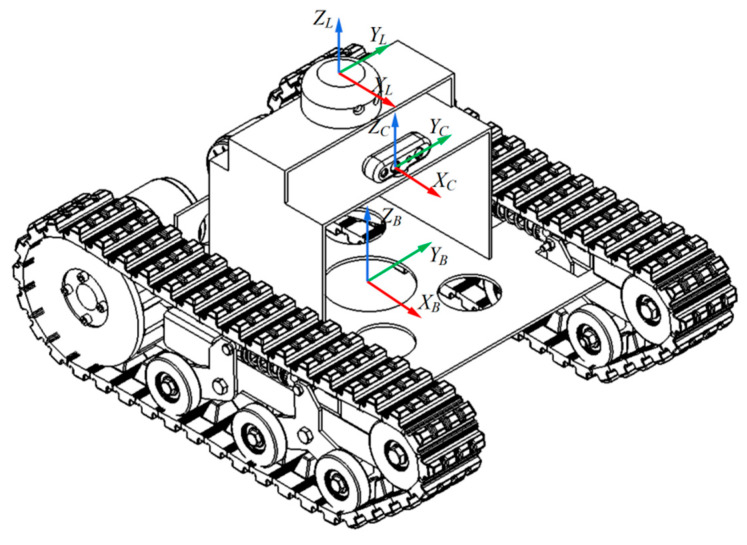
Positional relationship between chassis and sensors.

To achieve effective fusion of multi-sensor data, this study established a standardized coordinate system framework. The chassis coordinate system is defined as the reference coordinate system, with its origin located at the chassis center point at a height above the ground. The LiDAR coordinate system has its origin at the LiDAR scanning center at a height above the ground. The camera coordinate system has its origin at the RGB camera optical center at a height above the ground. All proposed coordinate systems follow the convention where the *x*-axis points toward the vehicle’s forward direction, the *y*-axis points toward the vehicle’s left side, and the *z*-axis points vertically upward in accordance with the right-hand rule. The LiDAR is mounted directly above the origin of the chassis coordinate frame, while the camera is spatially offset relative to the LiDAR. The precise relative pose between the two sensors is obtained via joint extrinsic calibration. The arrangement of the mobile chassis and the two sensors is shown in Figure 3.

#### 2.2.2. Spatial Fusion

**Figure 4:**
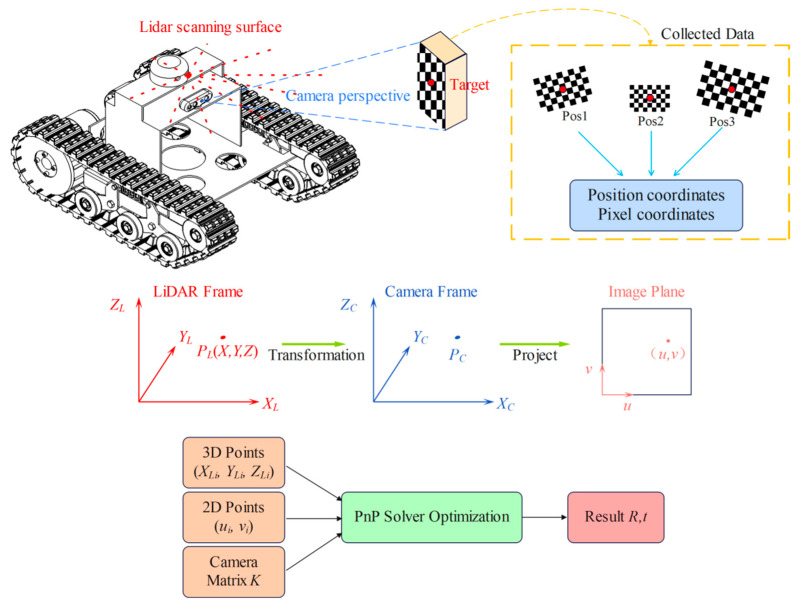
Schematic diagram of extrinsic parameter calibration.

Camera intrinsic parameter calibration was performed using Zhang’s calibration method [38], employing the Camera Calibrator toolbox in MATLAB (2021b). The checkerboard grid used featured 8 × 5 inner corner points, with each square having a side length of 27 mm. As shown in Figure 4, checkerboard images were captured under different poses, and the camera’s focal length, principal point coordinates, radial distortion coefficients, and tangential distortion coefficients were calculated using functions provided by the OpenCV (3.3.1) library. The calibration process was repeated multiple times, and average values were taken to improve accuracy. The camera intrinsic parameter matrix is shown in Equation (1).

(1) $$K=\left[\begin{array}{ccc} f_{x} & 0 & c_{x}\\ 0 & f_{y} & c_{y}\\ 0 & 0 & 1 \end{array}\right]$$

Extrinsic parameter calibration between the camera and LiDAR is a crucial step for achieving multi-sensor data fusion. As illustrated in Figure 4, this study proposes an extrinsic calibration method suitable for 2D LiDAR and camera systems. The method first obtains the pixel coordinates of the calibration board’s center point and its coordinates in the LiDAR coordinate system, then employs the PnP (Perspective-n-Point) algorithm to accurately solve the spatial pose relationship of the camera relative to the LiDAR.

The essence of extrinsic calibration is to determine the rigid body transformation relationship between the LiDAR coordinate system and the camera coordinate system. The proposed 2D LiDAR-camera extrinsic calibration method takes pixel coordinates and radar coordinates as inputs, first converting the calibration board center point from the LiDAR coordinate system to the camera coordinate system. The transformation relationship between the two systems is shown in Equation (2).

(2) $$\left[\begin{array}{c} X_{C}\\ Y_{C}\\ Z_{C} \end{array}\right]=R\left[\begin{array}{c} X_{L}\\ Y_{L}\\ Z_{L} \end{array}\right]+t$$

where R is a 3 × 3 rotation matrix, t is a 3 × 1 translation vector, ${\left[\begin{array}{ccc} X_{C} & Y_{C} & Z_{C} \end{array}\right]}^{T}$ represents the coordinates of the target point in the camera coordinate system, and ${\left[\begin{array}{ccc} X_{L} & Y_{L} & Z_{L} \end{array}\right]}^{T}$ represents the coordinates of the target point in the radar coordinate system.

The coordinates of the calibration board center point are converted from the camera coordinate system to the pixel coordinate system through the transformation shown in Equation (3). The relationship between the camera coordinate system and pixel coordinate system has been extensively studied [39,40] and will not be elaborated upon in this paper.

(3) $$\left[\begin{array}{c} u\\ v\\ 1 \end{array}\right]=\left[\begin{array}{c} f_{x}\frac{X_{C}}{Z_{C}}+c_{x}\\ f_{y}\frac{Y_{C}}{Z_{C}}+c_{y}\\ 1 \end{array}\right]$$

The conversion process from the radar coordinate system to the pixel coordinate system is thus expressed as:

(4) $$s\left[\begin{array}{c} u\\ v\\ 1 \end{array}\right]=K\left[\begin{array}{cc} R & t \end{array}\right]\left[\begin{array}{c} X_{L}\\ Y_{L}\\ \begin{array}{c} Z_{L}\\ 1 \end{array} \end{array}\right]=\left[\begin{array}{ccc} f_{x} & 0 & c_{x}\\ 0 & f_{y} & c_{y}\\ 0 & 0 & 1 \end{array}\right]\left[\begin{array}{cc} R & t \end{array}\right]\left[\begin{array}{c} X_{L}\\ Y_{L}\\ \begin{array}{c} Z_{L}\\ 1 \end{array} \end{array}\right]$$

where s is the scale factor and K is the camera intrinsic parameter matrix.

For a given set of n calibration point pairs ${\left\{\left(u_{i},v_{i},X_{Li},Y_{Li},Z_{Li}\right)\right\}}_{i=1}^{n}$, as shown in Equation (5), the objective of the PnP problem is to solve for the optimal rotation matrix R and translation vector t.

(5) $$\underset{R,t}{\min}\sum_{i=1}^{n}{\left\|P_{i}-\pi \left(K,R,t,P_{Li}\right)\right\|}^{2}$$

where $P_{i}={\left[u_{i},v_{i}\right]}^{T}$ is the i-th pixel point, $P_{Li}={\left[X_{Li},Y_{Li},Z_{Li}\right]}^{T}$ is the three-dimensional coordinates of the i-th point in the LiDAR coordinate system, and $\pi \left(K,R,t,P_{Li}\right)$ is the function that projects the i-th point to the pixel coordinate system through transformation parameters (R, t).

The position of the camera in the LiDAR coordinate system is calculated as:

(6) $$C\;=\;-R^{T}t$$

Based on the aforementioned mathematical model and algorithm workflow, precise extrinsic calibration between the camera and 2D LiDAR can be achieved. This method establishes a complete coordinate transformation chain from the LiDAR coordinate system to the camera coordinate system and then to the pixel coordinate system. By utilizing the PnP algorithm to solve for the rotation matrix R and translation vector t, the relative position of the camera with respect to the 2D LiDAR is computed, thereby achieving precise alignment of data from both sensors within a unified coordinate system.

#### 2.2.3. Temporal Fusion

In the camera-LiDAR extrinsic calibration process, ensuring temporal consistency between the two sensor data streams is a critical prerequisite for obtaining accurate calibration results. In multi-sensor fusion systems, temporal alignment strategies are primarily categorized into synchronous fusion and asynchronous fusion [41]. Since pomegranate orchard tree trunks serve as navigation reference targets with fixed positions and the mobile platform experiences minimal positional changes over short time periods, while the navigation system requires rapid response and should avoid excessive data waiting times, an asynchronous fusion strategy is more suitable for pomegranate orchard navigation environments.

**Figure 5:**
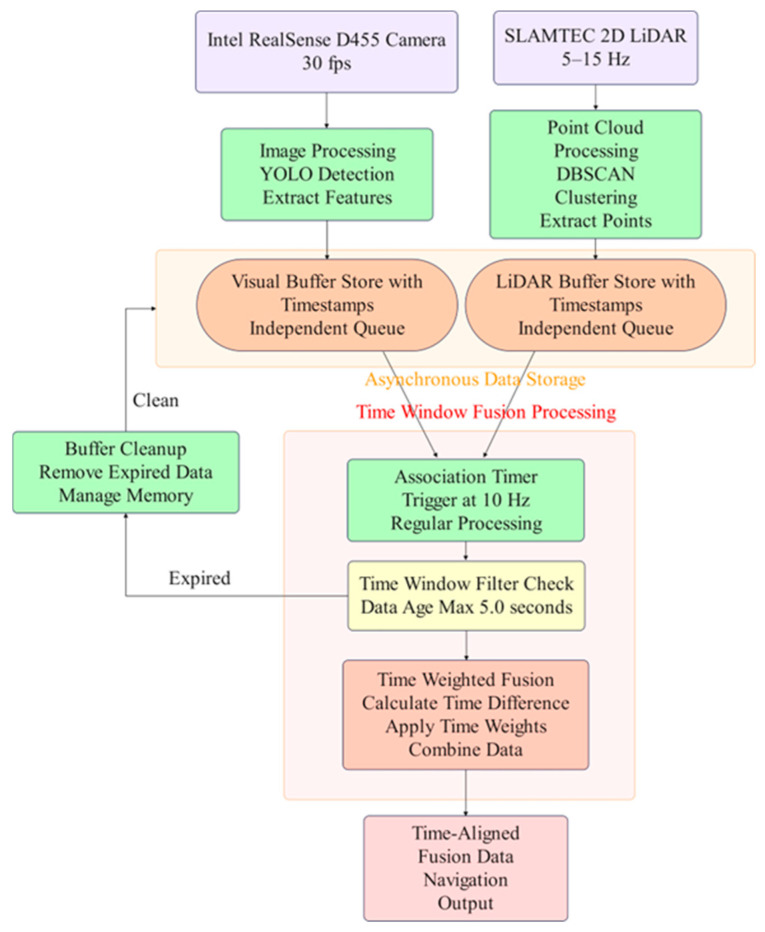
Temporal fusion model for monocular camera and 2D LiDAR.

Due to the Intel RealSense D455 camera outputting RGB images at 30 fps and the SLAMTEC 2D LiDAR operating at a scanning frequency of 5–15 Hz, their respective data arrival intervals are 33.3 ms and 67–200 ms. Without appropriate temporal fusion processing, this would lead to incorrect calibration point correspondences, thereby affecting the solution accuracy of the PnP algorithm and producing erroneous spatial associations. As shown in Figure 5, a parallel approach is adopted to independently process Intel RealSense D455 camera data and SLAMTEC 2D LiDAR data, where visual features are extracted through YOLO detection algorithms and point cloud data undergoes DBSCAN clustering processing. Each sensor data stream is stored in independent circular buffers that retain timestamp information, eliminating the need for strict synchronization constraints. The system employs a 10 Hz timing-triggered data association mechanism between the two sensors, periodically retrieving data from both buffers within a 5 s time window, ensuring computational efficiency while guaranteeing access to fresh data. The system automatically removes expired data beyond the time window, ensuring the use of consistent stored data and preventing data accumulation.

Each camera frame is paired with the temporally nearest 2D LiDAR scan. Within each trunk detection bounding box, we compute the nearest distance between the pixel rays and the raw LiDAR point clusters; if both a distance threshold and a directional-consistency check are satisfied, the cluster is accepted and its centroid is projected onto the *Z* = 0 plane of the mobile chassis coordinate frame *B* to obtain a trunk point. All trunk points are then fitted with RANSAC to obtain the row-direction navigation line. The method fuses directly on raw pixels and raw measurements, facilitating real-time operation.

### 2.3. Algorithm Framework

**Figure 6:**
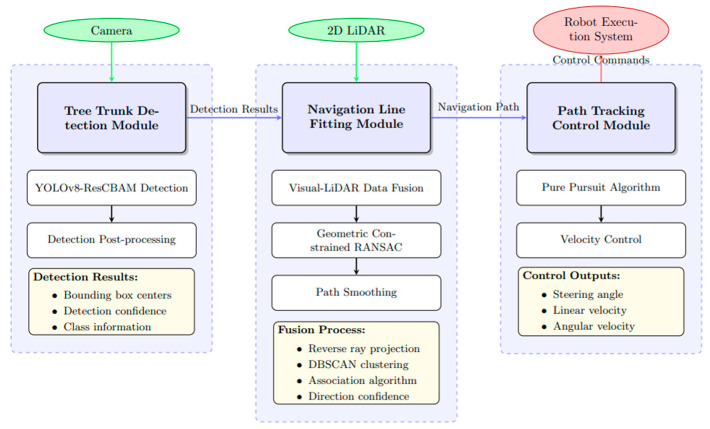
Vision-LiDAR Fusion Orchard Navigation System Architecture.

The proposed vision and LiDAR fusion-based orchard navigation system adopts a modular design architecture, primarily comprising three components: trunk detection module, navigation line extraction module, and path tracking control. The overall system architecture is illustrated in Figure 6.

System architecture showing the data flow between three core modules. The Tree Trunk Detection Module processes camera images and outputs detection results to the Navigation Line Fitting Module, which fuses these with LiDAR data to generate navigation paths. The Path Tracking Control Module then converts these paths into control commands for the robot execution system.

### 2.4. YOLOv8-ResCBAM-Based Trunk Detection

Trunk detection in pomegranate orchard environments faces numerous technical challenges. Pomegranate juvenile trees have relatively small trunk diameters and exhibit low contrast characteristics against complex backgrounds. Simultaneously, branch and leaf occlusion, weed interference, and varying lighting conditions further increase detection difficulty and are prone to causing detection confusion.

#### 2.4.1. YOLOv8-ResCBAM Model Applicability Analysis

To select an appropriate detection method for pomegranate orchard environments, this study conducted comparative analysis of mainstream object detection technologies. Traditional image processing methods, while computationally efficient, lack robustness under complex lighting and occlusion conditions. The deep learning-based YOLOv8 model achieves a good balance between speed and accuracy, but still has limitations in detecting small and occluded targets [42]. Considering the aforementioned issues comprehensively, this study selected the YOLOv8-ResCBAM model proposed by Ju et al. [43] as the fundamental architecture for trunk detection. The model is an efficient enhancement of the YOLOv8 architecture that integrates residual connections and a Convolutional Block Attention Module (CBAM). In orchard environments where trunks are small and planting density is high, traditional object detection methods often fail under background clutter or illumination changes. Leveraging improved feature extraction and attention, YOLOv8-ResCBAM can effectively discriminate fruit-tree trunks from complex backgrounds and remain robust to occlusions and illumination variations. Consequently, the model substantially enhances feature representation while maintaining computational efficiency, thereby providing high-precision trunk detections for the subsequent navigation line extraction.

YOLOv8-ResCBAM effectively mitigates the gradient vanishing problem in deep networks through ResBlock structures, improving the retention capability for small target features. In densely planted pomegranate orchard environments, this characteristic helps accurately identify slender tree trunks and reduces missed detections caused by feature loss. The Convolutional Block Attention Module (CBAM) comprises two sub-modules: channel attention and spatial attention. The channel attention mechanism enhances the model’s perception capability for trunk texture features by learning importance weights of different feature channels, effectively suppressing interference from background noise such as weeds. The spatial attention mechanism improves recognition capability for partially occluded tree trunks by focusing on key regions within feature maps.

#### 2.4.2. YOLOv8-ResCBAM Training and Implementation

A large amount of image data was collected in pomegranate orchard environments, totaling 4150 images after data augmentation, with 70% allocated for training, 20% for validation, and 10% for testing. Tree trunks in each image were annotated using the Labelimg (1.7.1) annotation tool. The training parameters were configured as follows:

(1) SGD optimizer with an initial learning rate of 1 × 10^−2^, weight decay of 5 × 10^−4^, and momentum of 0.937.

(2) Input image size of 640 × 640, batch size of 8, training for 200 epochs. To prevent overfitting, a cosine annealing learning rate scheduling strategy was employed.

(3) Data augmentation techniques including random scaling, random rotation, color adjustment (HSV), and horizontal flipping were applied during the training process.

The detection results output by the trained model include bounding box coordinates, confidence scores, and class labels. The center point coordinates of the bounding box $\left(cx,cy\right)$ are extracted as shown in Equation (6), and detection results with confidence scores above the threshold (τ = 0.5) are published as ROS topics for subscription by downstream modules.

(7) $$cx=\frac{x_{1}+x_{2}}{2},cy=\frac{y_{1}+y_{2}}{2}$$

### 2.5. Vision and LiDAR Fusion-Based Navigation Line Fitting

#### 2.5.1. DBSCAN Trunk Clustering Algorithm

In the dense planting environment of pomegranate orchards with trunk diameters of 5–15 cm and plant spacing of 1–2 m, point cloud data acquired by 2D LiDAR exhibits characteristics of roughly row-wise arrangement. However, variations in plant spacing and local occlusions may result in incomplete or irregular point clouds. These characteristics render traditional clustering methods based on shape assumptions ineffective, making it difficult to effectively distinguish actual tree trunks from background noise44 [44]. Therefore, the DBSCAN (Density-Based Spatial Clustering of Applications with Noise) algorithm, which performs clustering based on density connectivity, is employed for LiDAR point cloud clustering. This algorithm is suitable for processing noisy data and irregularly shaped clusters, making it extremely effective for trunk detection in orchard environments [45].

Raw measurements are first limited to the [1,1.5] m range window according to the orchard row spacing, removing near- and far-field non–tree-row interference. Following the scan order, a median filter with window width *k* = 5 is applied to the point-cloud ranges $r_{i}$ to obtain $\bar{r_{i}}$. When $\left|r_{i}-\bar{r_{i}}\right|\;>\;r_{med}=0.15\;m$ holds, the sample is regarded as a spike and replaced with $\bar{r_{i}}$. This step effectively suppresses non–tree-row clutter and sporadic outliers, providing a cleaner point set for DBSCAN.

**Figure 7:**
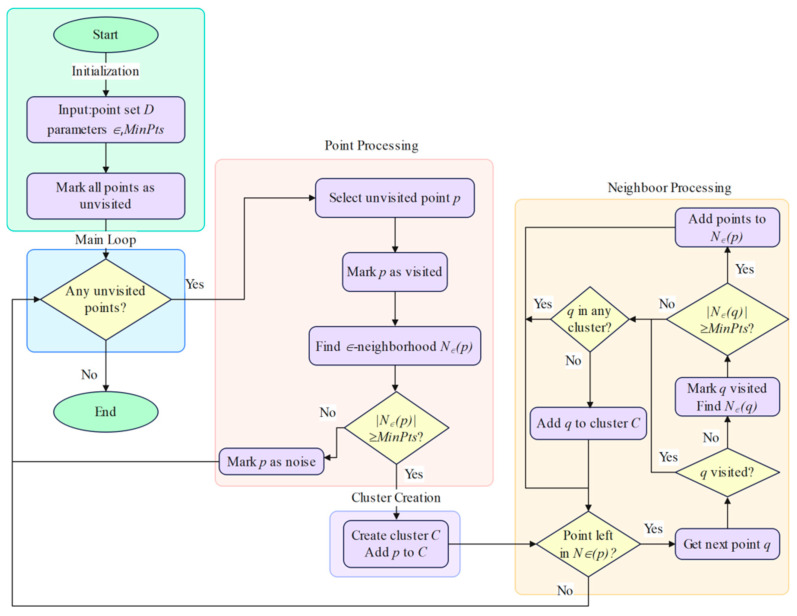
DBSCAN algorithm flowchart.

The DBSCAN algorithm workflow for pomegranate orchard environments is illustrated in Figure 7. The algorithm classifies a given point set $D=\{p_{1},p_{2},\ldots ,p_{n}\}$ into three categories:

Core points: points that have at least MinPts points within their ϵ neighborhood radius;

Border points: points that have fewer than MinPts points within their ϵ neighborhood radius but are within the ϵ neighborhood radius of some core point;

Noise points: points that are neither core points nor border points.

The performance of the DBSCAN algorithm is primarily determined by two key parameters: the neighborhood radius ϵ and the minimum number of points MinPts. Parameter selection must comprehensively consider the geometric characteristics of pomegranate orchards, LiDAR specifications, and clustering accuracy requirements.

The selection of neighborhood radius ϵ requires balancing clustering completeness and accuracy. An excessively small ϵ value would cause a single tree to be segmented into multiple clusters, while an excessively large ϵ value might merge adjacent trees into one cluster. The MinPts parameter affects the noise resistance and completeness of clustering. The selection of this parameter is based on the number of trunk reflection points that LiDAR can acquire at different distances. Based on the geometric constraints of pomegranate orchards, the theoretical ranges of ϵ and MinPts values can be determined through the following analysis:

Pomegranate juvenile trees have diameters of 5–15 cm, with plant spacing of 1–2 m, row spacing of 2–3 m, and LiDAR angular resolution of 0.225°. To ensure that point clouds from individual tree trunks are not over-segmented, the minimum value of ϵ should be greater than 1.5 times the minimum trunk diameter, and the maximum value should be less than half the minimum plant spacing, i.e., $\epsilon \;\in \;\left[0.075,0.5\right]$.

Based on LiDAR technical specifications and trunk geometric characteristics, the theoretical point count estimation for a single trunk with 5 cm diameter at 1.5 m detection distance is shown in Equation (8).

(8) $$N_{P}=\frac{D}{d_{res}}=\frac{D\;\times \;360}{2\pi r\;\times \;0.225}\approx 8$$

where D is the trunk diameter, $d_{res}$ is the radar linear resolution, and r is the detection distance.

Considering practical factors such as occlusion and reflection intensity variations, setting $MinPts\in \left[3,5\right]$ can effectively filter noise points while ensuring clustering completeness. Based on the above, the DBSCAN neighborhood radius ϵ and the minimum number of points MinPts are derived from the LiDAR’s angular resolution and the desired linear resolution corresponding to the expected trunk diameter, selecting a target scale suited to densely planted inter-row spacing. This configuration strikes a balance between noise suppression and cluster integrity.

#### 2.5.2. Vision and LiDAR Data Association

##### Line-of-Sight and LiDAR Plane Intersection Calculation

This study employs a loosely coupled sensor fusion method to associate and fuse visual detection results with 2D LiDAR data. The vision system provides pixel coordinates of tree trunks, while LiDAR provides accurate distance information. Through joint calibration of multiple sensors to the chassis reference coordinate system, this study performs processing and computation for vision and LiDAR data fusion.

This study proposes a reverse ray projection algorithm that differs from traditional point cloud-to-image plane projection methods. The algorithm projects pixel coordinates from visual detection into three-dimensional rays and computes their intersections with LiDAR scanning planes to achieve data association.

The pixel point $\left(u,v\right)$ from visual detection is converted to a ray direction in the camera coordinate system:

(9) $$\left[\begin{array}{c} x_{norm}\\ y_{norm}\\ 1 \end{array}\right]=\left[\begin{array}{c} \frac{u-c_{x}}{f_{x}}\\ \frac{v-c_{y}}{f_{y}}\\ 1 \end{array}\right]$$

The camera principal point $\left(c_{x},c_{y}\right)$ and focal length $\left(f_{x},f_{y}\right)$ are obtained through camera intrinsic parameter calibration.

The ray direction in the reference coordinate system is then calculated as:

(10) $${\vec{ray}}_{dir}=R_{cb}\left[\begin{array}{c} x_{norm}\\ y_{norm}\\ 1 \end{array}\right]$$

The LiDAR plane can be expressed as:

(11) $$a_{l}X_{b}+b_{l}Y_{b}+c_{l}Z_{b}+d_{l}=0$$

where $\left(a_{l},b_{l},c_{l}\right)$ is the plane normal vector and $d_{l}$ is the distance from the plane to the origin.

The intersection point between the ray and plane can be obtained by solving the following equation:

(12) $$t=-\frac{a_{l}\left(O_{x}\right)+b_{l}\left(O_{y}\right)+c_{l}\left(O_{z}\right)+d_{l}}{a_{l}\left(D_{x}\right)+b_{l}\left(D_{y}\right)+c_{l}\left(D_{z}\right)}$$

where $\left(O_{x},O_{y},O_{z}\right)$ is the ray origin and $\left(D_{x},D_{y},D_{z}\right)$ is the ray direction.

The intersection coordinates are:

(13) $$P_{intersect}=\left[\begin{array}{c} O_{x}\\ O_{y}\\ O_{z} \end{array}\right]+t\left[\begin{array}{c} D_{x}\\ D_{y}\\ D_{z} \end{array}\right]$$

##### Association Algorithm

**Figure 8:**
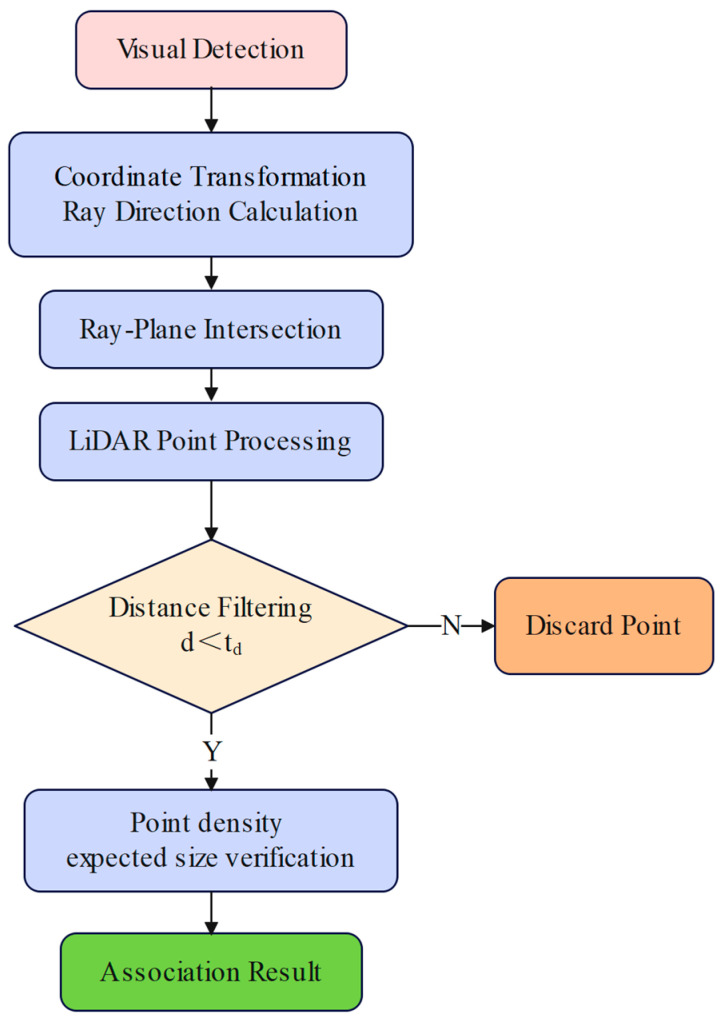
Association algorithm flowchart.

This paper proposes a vision and LiDAR data association algorithm that matches visually detected trunk points with LiDAR-clustered trunk point clouds. Unlike traditional data association methods that employ point cloud projection onto image planes, this study proposes a vision and LiDAR data association algorithm based on reverse ray projection. The core principle involves calculating the minimum distance between visual rays and LiDAR points and setting thresholds for association. The algorithm workflow is illustrated in Figure 8.

Given a visual ray with origin O and direction $\vec{d}$, and a LiDAR point P, the minimum distance from the point to the ray is:

(14) $$dist\left(P,\mathrm{ray}\right)=\left|\vec{OP}-\left(\vec{OP}\cdot \vec{d}\right)\vec{d}\right|$$

where $\vec{OP}$ is the vector from the ray origin to point P, $\left(\vec{OP}\cdot \vec{d}\right)$ is the projection length of $\vec{OP}$ along the ray direction, and $\left(\vec{OP}\cdot \vec{d}\right)\vec{d}$ is the projection vector.

The proposed vision and LiDAR data association algorithm back-projects visual detections in the pixel coordinate system into three-dimensional rays in the reference coordinate system through camera intrinsic parameter matrix and distortion correction. Each ray extends from the camera center toward the detected target. The algorithm calculates the minimum distance from point clouds to rays and treats laser points with distances smaller than the preset threshold $\tau_{d}$ as matching candidate points. To ensure detection effectiveness, spatial neighborhood point density analysis and consistency verification based on expected trunk dimensions are performed. Finally, the DBSCAN clustering algorithm is employed to merge multiple detections belonging to the same trunk, generating precise trunk positions through centroid calculation.

**Figure 9:**
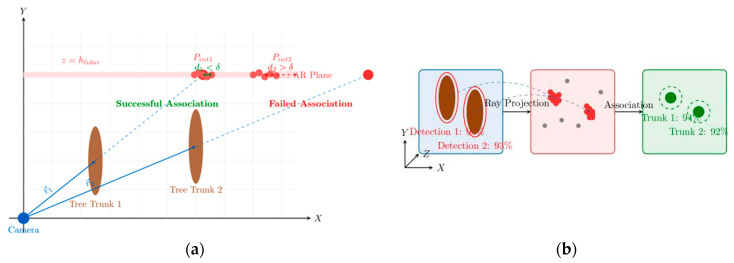
Schematic diagram of the association algorithm: (**a**) Geometric principle—the camera ray intersects the LiDAR height plane ({z=h}_{lidar}); items within the distance threshold \delta are shown in green (successful association), whereas those beyond \delta are in red (failed association). (**b**) Association process—the blue dashed line denotes the reverse ray projection from the camera optical center through each 2D trunk detection; LiDAR points close to this ray are highlighted in red as candidates, gray dots indicate background returns, and the finally associated trunks are shown in green.

As shown in Figure 9, Figure 9a illustrates the geometric principle of the association algorithm: the ray casting method projects visual detections into 3D space, and association succeeds when LiDAR points satisfy the distance threshold and density constraints of the projection geometric boundaries. Figure 9b shows the three stages of vision and LiDAR association: the visual detection stage that generates candidate regions with confidence scores, LiDAR point cloud data acquisition, and the final associated point clusters after processing.

#### 2.5.3. Navigation Line Fitting

##### Geometric Constraint Navigation Line Fitting

After obtaining the associated points, the RANSAC (Random Sample Consensus) algorithm is employed to fit fruit tree rows. The RANSAC algorithm can robustly fit models in the presence of numerous outliers, making it suitable for addressing scenarios with weeds and irregular arrangements in orchard environments [46]. Considering detection errors and planting irregularities comprehensively, the distance threshold for the RANSAC algorithm is set to τ = 0.15 m. Given that interference from weeds, support poles, and other factors in orchard environments reduces the inlier ratio, the maximum number of iterations is set to 100 to ensure algorithm robustness.

**Figure 10:**
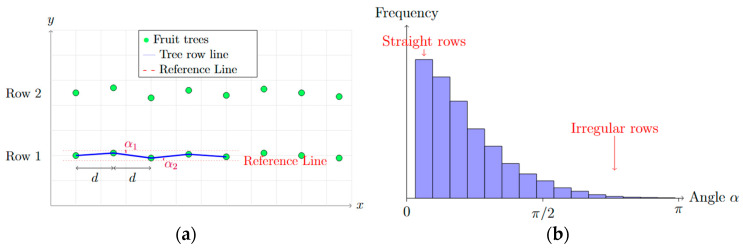
Geometric constraint navigation line fitting: (**a**) Fruit tree angle analysis; (**b**) Angle histogram.

Although the conventional RANSAC algorithm exhibits good robustness to noise and outliers in orchard environments, its reliance on random sampling can lead to unstable fitting results under complex conditions. In particular, when tree planting is irregular and interference is heavy, it remains susceptible to errors, making it difficult to ensure fitting accuracy [47]. To improve fitting stability and accuracy, this paper introduces a Geometric Constraint Algorithm (GCA). Field investigations reveal that fruit tree rows in pomegranate orchards are typically planted along the same direction to facilitate orchard management. As shown in Figure 10, this study analyzes the geometric relationships between fruit trees based on orchard characteristics and constructs angle histograms to identify the main navigation direction for constraining the fitting process. Compared with the traditional RANSAC method, the geometric-constraint method (GCA) introduces a directional-consistency constraint that prioritizes a fitting direction aligned with the orchard’s planting pattern, thereby effectively improving fitting accuracy and stability. When facing interfering data and environmental variations, it markedly reduces the fitting error. By fully exploiting the regularity of tree rows, the method avoids sampling-induced errors inherent to RANSAC and substantially enhances both robustness and accuracy.

The directional angles between all trunk point pairs $\left(p_{i},p_{j}\right)$ are analyzed to construct a directional histogram:

(15) $$\theta_{ij}=atan2\left(p_{jy}-p_{iy},p_{jx}-p_{ix}\right)$$

Angles are normalized to the $\left[0,\pi \right)$ range to avoid directional differences:

(16) $$\theta_{norm}=\theta_{ij}\;mod\pi$$

Histogram statistics are performed on the normalized angles with intervals of π/18, and the weight for each interval is:

(17) $$w_{k}=\sum_{\left(i,j\right)\epsilon bin_{k}}{conf}_{i}\cdot {conf}_{j}\cdot \exp\left(-d_{ij}/\sigma \right)$$

where ${conf}_{i}$ and ${conf}_{j}$ are the confidence scores of the points, $d_{ij}$ is the point pair distance, and σ is the distance weight coefficient.

The histogram interval with the maximum weight is identified as the main direction:

(18) $$\theta_{main}=\frac{\sum_{\theta_{k}\epsilon bin_{max}}\theta_{k}\cdot w_{k}}{\sum_{\theta_{k}\epsilon bin_{max}}w_{k}}$$

Based on the main direction analysis, directional confidence λ is defined to quantify the reliability of the current direction estimation:

(19) $$\lambda =\min\left(1.0,\frac{n_{inliers}}{n_{min}}\cdot \frac{l_{fit}}{l_{min}}\cdot \frac{w_{max}}{\sum w_{k}}\right)$$

where $n_{inliers}$ is the number of inliers, $n_{min}$ is the minimum required number of inliers, $l_{fit}$ is the fitted line length, $l_{min}$ is the minimum required length, $w_{max}$ is the maximum weight, and $\sum w_{k}$ is the total weight.

When the confidence $\lambda >\lambda_{threshold}=0.6$, the current estimated direction is used to constrain subsequent fitting:

(20) $$\theta_{constrained}=\theta_{current}$$

Under geometric constraints, the line slope is fixed and only the intercept is estimated:

(21) $$y=\tan\left(\theta_{constrained}\right)\cdot x+b$$

where the intercept b is:

(22) $$b=\frac{1}{n}\sum_{i=1}^{n}\left(y_{i}-\tan\left(\theta_{constrained}\right)\cdot x_{i}\right)$$

After obtaining the left and right row fitting lines, the center navigation line is generated by calculating the midpoints of the two lines. Assuming the left and right row equations are:

(23) $$\left\{\begin{array}{c} y_{L}=m_{L}x+b_{L}\\ y_{R}=m_{R}x+b_{R} \end{array}\right.$$

The center line equation is:

(24) $$y_{C}=\frac{m_{L}+m_{R}}{2}x+\frac{b_{L}+b_{R}}{2}$$

##### Kalman Filter Smoothing

To reduce navigation line jumps caused by noise, a Kalman filter is employed to smooth the fitting parameters and reduce noise effects [48]. For each row of fruit trees on the left and right sides, the state vector is $x={\left[m,b\right]}^{T}$, representing the slope and intercept parameters of the fitted line for that tree row, respectively.

Considering the fixed nature of fruit tree rows in orchard environments, navigation line parameters change slowly over short periods, thus a constant velocity model is adopted:

(25) $$x_{k}=x_{k-1}+w_{k-1}$$

The observation equation is:

(26) $$z_{k}=x_{k}+v_{k}$$

where $w_{k-1}$ and $v_{k}$ are the process noise and measurement noise, respectively.

The prediction steps are:

(27) $$x_{k|k-1}=x_{k-1|k-1}$$

(28) $$P_{k|k-1}=P_{k-1|k-1}+Q_{k-1}$$

The update steps are:

(29) $$K_{k}=P_{k\left|k-1\right.}{\left(P_{k\left|k-1\right.}+R\right)}^{-1}$$

(30) $$X_{k\left|k\right.}=X_{k\left|k-1\right.}+K_{k}(Z_{k}-X_{k\left|k-1\right.})$$

(31) $$P_{k|k}=\left(I-K_{k}\right)P_{k|k-1}$$

where P is the state covariance matrix, Q is the process noise covariance matrix, R is the measurement noise covariance matrix, and K is the Kalman gain. According to the characteristics of pomegranate orchard environments, the process noise covariance is set to $Q=diag\left(\left[0.01,0.05\right]\right)$ to reflect the relative stability of navigation line parameters, where slope changes are smaller than intercept changes. The measurement noise covariance $R=diag\left(\left[0.1,0.2\right]\right)$ is determined based on statistical analysis of RANSAC fitting accuracy. From the weighted angle histogram in Section Geometric Constraint Navigation Line Fitting the principal row-direction angle $\theta_{main}$ is obtained, and the vehicle’s lateral offset from the row line is computed. The heading and lateral offset, after first-order Kalman filtering, are fed to the Pure Pursuit algorithm to generate linear and angular velocity commands. This interface decouples perception from control and effectively suppresses command jitter under short-term occlusions or abrupt illumination changes.

### 2.6. Experimental Methods

**Figure 11:**
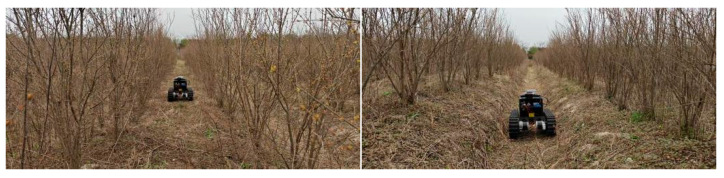
Experimental scenarios.

To validate the effectiveness and accuracy of the proposed vision and 2D LiDAR fusion-based orchard navigation system, this paper conducted sensor calibration experiments, visual detection performance evaluation experiments, data association verification experiments, geometric constraint navigation line fitting simulation experiments, and navigation line tracking comparison experiments. Experiments were conducted at the Anhui Pomegranate Germplasm Resource Nursery in Bengbu, Anhui Province, China (117°4′15.764880″ E, 32°58′53.022720″ N). The site exhibits typical densely planted pomegranate-orchard characteristics and includes trees at multiple growth stages; this study focused primarily on 1–3-year-old trees with trunk diameters of 5–15 cm, an in-row spacing of 1–2 m, and a row spacing of 2–3 m. The orchard layout of the test area is shown in Figure 11. Representative operating scenarios included straight rows, slight lateral offset, and cross-slope operation. Weather and illumination conditions were sunny, overcast, dusk, strong direct light, and backlit. In each scenario, one operating row was covered; each single-row segment contained 25–32 trees, and every scenario was repeated 10 times to assess stability and repeatability. In total, approximately 1400 trees were recorded. Throughout the experiments, the sensor mounting configuration and algorithm parameters were kept uniform.

(1) The sensor calibration experimental methodology was as follows: Zhang’s calibration method was employed for camera intrinsic parameter calibration, capturing 20 images of different poses using an 8 × 5 checkerboard (27 mm side length); the PnP algorithm was used for camera and 2D LiDAR extrinsic parameter calibration, obtaining multiple sets of calibration board center point positions in the radar coordinate system and pixel coordinates. Evaluation metrics included reprojection error and calibration accuracy.
(2) The visual detection performance evaluation experimental methodology was as follows: 4150 images were collected under different lighting conditions (sunny, cloudy, dusk, strong light), divided into training, validation, and test sets at a 7:2:1 ratio, and the YOLOv8-ResCBAM model was used for trunk detection training and testing. Evaluation metrics included mAP50, recall rate, precision, and inference time.
(3) The data association verification experimental methodology was as follows: five groups of 20 m-long inter-row orchard environments were selected, each containing 25–32 fruit trees, with each group experiment repeated 10 times, using the reverse ray projection algorithm for vision and LiDAR data association. Evaluation metrics included association count and association success rate.
(4) The geometric constraint navigation line fitting simulation experimental methodology was as follows: six groups of simulation comparison experiments were designed with fruit tree point quantities ranging from 19 to 27, employing the proposed GCA and traditional RANSAC algorithm, respectively, for navigation line fitting. Evaluation metrics included fitting accuracy RMSE, inlier ratio, and computation time.
(5) The navigation-line tracking comparison was designed as follows. Five representative inter-row segments were selected in the orchard. Each segment (20 m) was manually surveyed to obtain the ground-truth centerline and included operating conditions such as straight rows, slight lateral offset, cross-slope, local occlusions, and non-uniform illumination. For each segment, 10 independent navigation trials were conducted at speeds from 0.5 to 2.0 m·s^−1^. Four navigation line extraction methods were compared: (i) single 2D LiDAR with RANSAC, (ii) single vision detection with RANSAC, (iii) DeepLab v3+ semantic segmentation with RANSAC, and (iv) the proposed fusion-based method. The mobile chassis position was recorded using a “taut-string–centerline-marking–tape-measurement” protocol. Specifically, a cotton string was stretched taut between the midpoints equidistant from the left and right rows at the two ends of the test segment to define the ground-truth centerline, and sampling stations were marked every 1 m along the string. A marker was mounted at the geometric center of the mobile chassis; after the vehicle passed, a measuring tape was used at each station to measure the perpendicular distance from the trajectory to the centerline, yielding the lateral deviation.

For the single 2D LiDAR + RANSAC method, the “2.5.1 DBSCAN trunk clustering algorithm” was used to obtain candidate points for the left and right rows, followed by separate line fittings with RANSAC to produce the two row lines and the center navigation line. For the single vision detection + RANSAC method, key pomegranate-tree targets were detected in images; low-confidence and out-of-range points were removed, and the remaining pixel points were projected onto the ground plane via the extrinsic parameters to form a point set for RANSAC fitting of the center navigation line. For the DeepLab v3+ semantic segmentation + RANSAC method, DeepLab v3+ (Backbone: ResNet-50, input 640 × 640) segmented ground and trunk background in each frame; the road mask underwent morphological opening/closing and Guo–Hall thinning to obtain a skeleton, on which RANSAC fitting was performed. The resulting centerline was then projected into the ground coordinate frame using the camera–LiDAR extrinsics to obtain the center navigation line. Evaluation metrics included the average lateral error, the lateral error RMS, and the navigation success rate (single-sided lateral error ≤ 10 cm).

## 3. Results

### 3.1. Calibration Experimental Results

**Figure 12:**
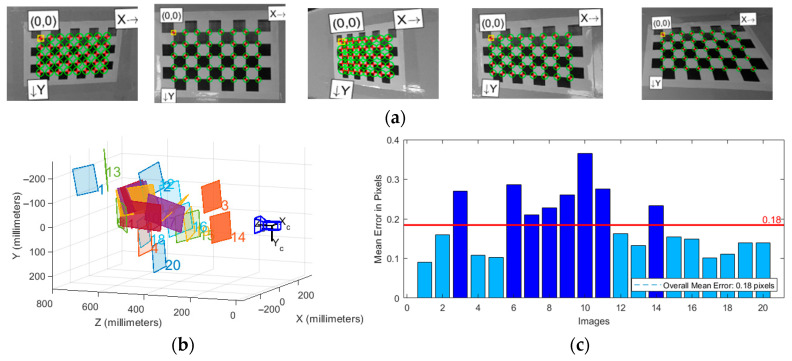
Camera intrinsic parameter calibration: (**a**) Partial images used for calibration; (**b**) Checkerboard and camera positions; (**c**) Image errors for calibration.

As shown in Figure 12, camera intrinsic parameter calibration was performed according to the monocular camera and 2D LiDAR data fusion method described in Section 2.2.

After calibration, the intrinsic parameter matrix *K* was obtained as shown in Equation (32), with an average reprojection error of 0.18 pixels.

(32) $$K\;=\left[\begin{array}{ccc} 639.3178 & 0 & 660.2825\\ 0 & 635.1858 & 363.30748\\ 0 & 0 & 1 \end{array}\right]$$

**Figure 13:**
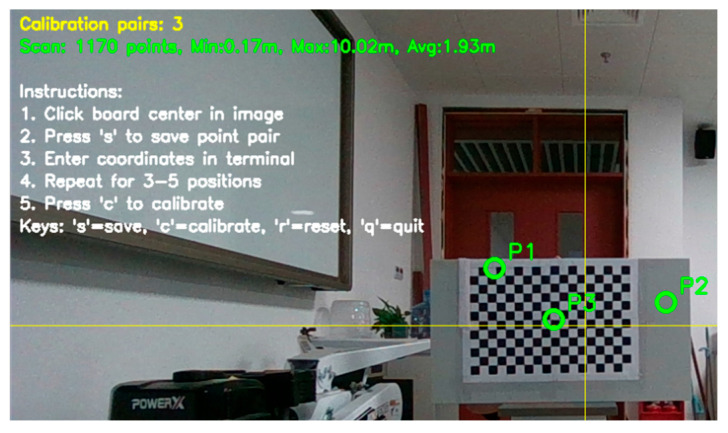
Camera and radar extrinsic parameter calibration.

In addition to camera intrinsic parameter calibration, it is necessary to determine the relative position of the camera on the mobile platform according to the camera and 2D LiDAR extrinsic parameter calibration method described in Section 2.2 to complete data fusion. The mobile platform is placed on a horizontal plane, with the 2D LiDAR coordinate system origin located 250 mm directly above the platform coordinate system origin. As shown in Figure 13, the radar coordinate system position and pixel coordinates of the calibration board center point are obtained.

Through calibration calculations on multiple sets of data, the three-dimensional coordinates $\left(t_{x},t_{y},t_{z}\right)$ of the camera relative to the 2D LiDAR were determined to be (0.075, 0.029, −0.096). Combining the results of camera intrinsic parameter calibration, camera and 2D LiDAR extrinsic parameter calibration, and temporal synchronization, the data fusion between camera and 2D LiDAR was completed, providing a consistent spatial reference for subsequent navigation algorithms.

### 3.2. Visual Detection Performance Evaluation

**Figure 14:**
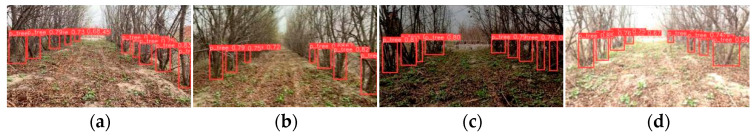
Tree trunk detection results under different environmental conditions: (**a**) Sunny; (**b**) Dusk; (**c**) Cloudy; (**d**) Strong light.

For different scenarios in pomegranate orchards, the detection results of the YOLOv8-ResCBAM model are shown in Figure 14.

As shown in Table 2, this paper conducted comparative analysis of different YOLOv8 model variants (each integrating different attention mechanisms) to evaluate their performance in detecting tree trunks in densely planted pomegranate orchards.

The results demonstrate that the YOLOv8-ResCBAM model outperforms the baseline YOLOv8 and other attention-enhanced variants. Comparison of various metrics reveals that YOLOv8-ResCBAM exhibits the highest mAP50 and recall rate, achieving 95.7% and 92.5%, respectively, indicating its superior capability in detecting tree trunks under high-density and occlusion environments. Although its precision (91.0%) is slightly lower than the highest-performing YOLOv8-SA (92.0%), it still maintains a high level, and the balance between precision and recall is more favorable for practical applications. The inference time of YOLOv8-ResCBAM is 15.36 ms, which, though longer than the baseline YOLOv8 (12.96 ms), remains within the acceptable range for real-time processing in autonomous navigation systems. Through comparison of various metrics, it is verified that YOLOv8-ResCBAM demonstrates the best comprehensive performance in enhancing feature extraction and focusing on salient regions. Particularly in complex environments with dense planting and the presence of weed and branch occlusion, the advantage of high recall rate is especially prominent, proving the applicability and superiority of this model in practical orchard application scenarios.

### 3.3. Data Association Results

**Figure 15:**
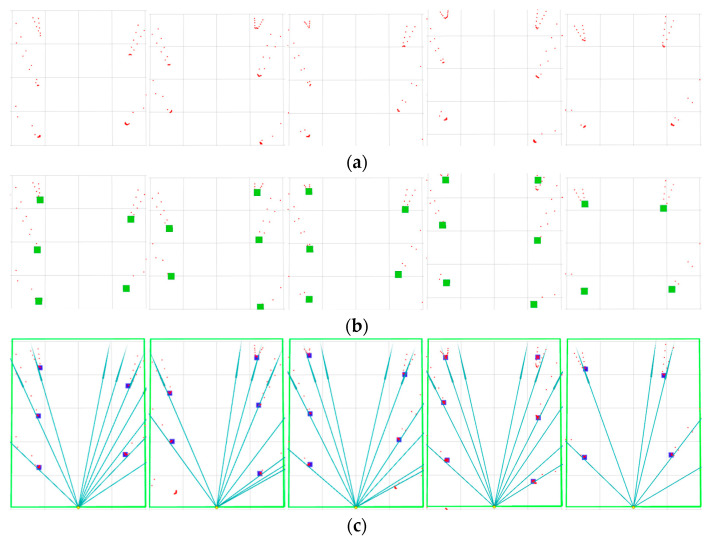
Data-association results. (**a**) 2D LiDAR point cloud—red dots are LiDAR returns. (**b**) LiDAR clustering—green solid squares mark cluster centroids (trunk hypotheses). (**c**) Visual–LiDAR as-sociation—blue solid lines are reverse ray projections from the camera optical center; blue solid squares denote LiDAR clusters that are successfully associated to the rays.

The experimental results of LiDAR clustering of pomegranate trees and their association with visual rays are shown in Figure 15. In Figure 15a, the red scatter points represent reflection points detected by LiDAR, and the point cloud distribution reflects the spatial structural characteristics of fruit tree rows. Figure 15b shows the results after clustering processing of LiDAR point clouds, where green points represent the identified fruit tree target center points after clustering, demonstrating that the clustering algorithm effectively extracted the positional information of fruit trees. Figure 15c displays the association effect between visual feature points and LiDAR clustered points, where blue rays represent rays emitted from the camera position toward target points, and blue points represent data points where visual rays successfully associated with laser clusters.

The results of five experimental groups, along with the average number of fruit trees and association counts, are presented in Table 3.

The experimental results demonstrate that the average association success rate reached 92.6%, indicating that the proposed vision and LiDAR data association algorithm possesses high accuracy. The association success rates for the five experimental groups under different fruit tree density environments (25–32 trees) consistently remained between 91.9 and 94.3%, with a standard deviation of 0.9%, validating the algorithm’s stability and adaptability, which meets the accuracy requirements for orchard navigation.

### 3.4. Geometric Constraint Navigation Line Fitting Simulation Results

**Figure 16:**
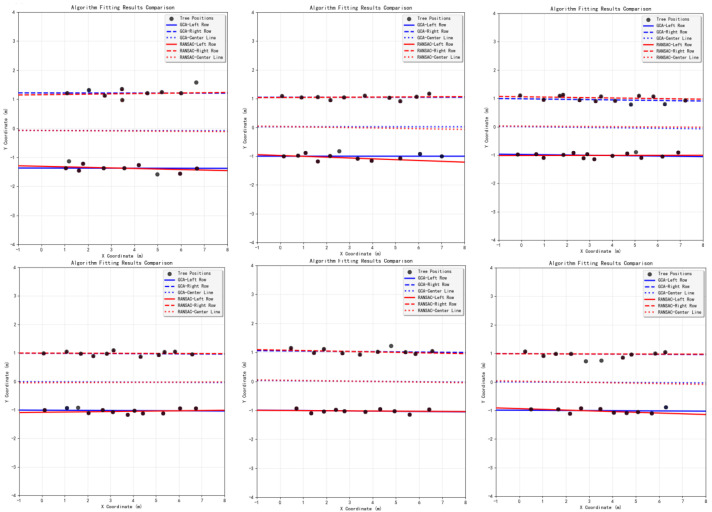
Experimental comparison between GCA and RANSAC algorithm.

As shown in Figure 16, a comparison of the visualization of fitting performance between the two algorithms in experiments is presented, where the blue line represents the fitting performance of the GCA and the red line represents the fitting performance of the RANSAC algorithm.

The detailed performance comparison results of the six experimental groups are shown in Table 4.

The experiments demonstrate that the proposed Geometric Constraint Algorithm exhibits significant advantages in orchard navigation line fitting tasks. Compared to the traditional RANSAC algorithm, GCA achieves an overall improvement of 2.4% in fitting accuracy. The average inlier ratio of GCA is 92.03%, which is 5.1% higher than RANSAC, demonstrating excellent noise resistance capability. GCA improves computational efficiency by 47.06%, meeting the requirements for real-time navigation.

### 3.5. Navigation Line Tracking Results

**Figure 17:**
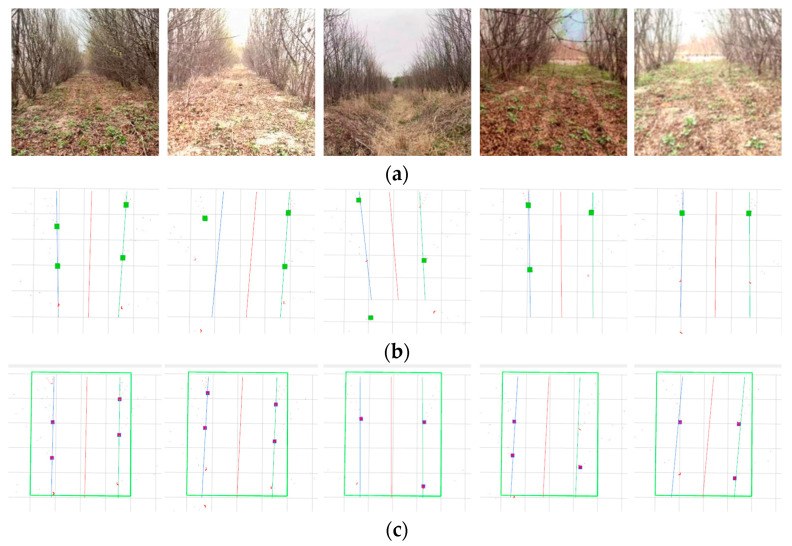

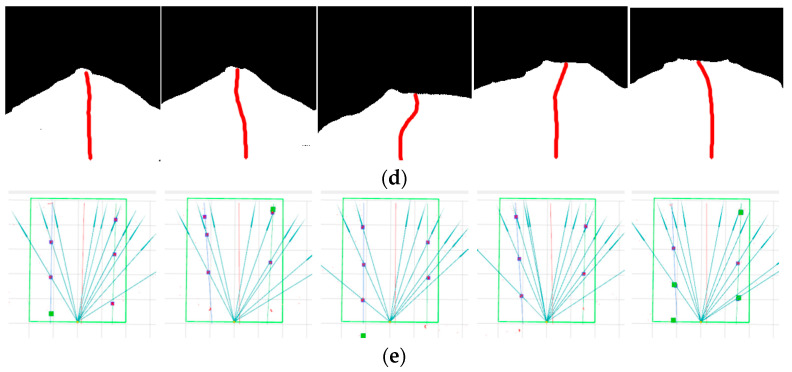
Experimental results of three navigation line extraction algorithms across five scenarios: (**a**) original camera images; (**b**) LiDAR-only fitting—red dots denote 2D LiDAR returns, green solid squares indicate LiDAR cluster centroids, and the cyan and red lines are the RANSAC-fitted left/right trunk-row lines and the navigation line; (**c**) vision-only fitting—blue squares mark 2D trunk detections, the green rectangle indicates the ROI, and the cyan and red lines are the RANSAC-fitted left/right trunk-row lines and the navigation line; (**d**) DeepLab v3+ semantic segmentation—black is background, white is the segmented ground region, and the red curve is the skeletonized center-line; (**e**) proposed fusion—blue solid lines with arrows denote reverse ray projections from the camera optical center; blue solid squares indicate LiDAR clusters successfully associated with the rays; green squares are the remaining unassociated clusters within the ROI; the cyan and red lines are as defined above.

The experimental results are shown in Figure 17, displaying the navigation line fitting results of three algorithms under five experimental conditions.

The experimental results are presented in Table 5, comparing the evaluation metrics of different navigation line extraction methods.

As shown in Figure 17, representative operating conditions—including straight rows, slight lateral offset, cross-slope, local occlusions, and non-uniform illumination—are presented together with the navigation-line fitting results of the four algorithms under the corresponding conditions. The results indicate that the proposed vision and 2D LiDAR fusion with a geometric constraint (GCA) improves both lateral deviation and navigation success rate over conventional methods. Although the gains relative to single-sensor and semantic-segmentation approaches are modest, they are crucial given the high occlusion, illumination variability, and small trunk size in orchards, helping ensure stable and efficient navigation for agricultural robots. Specifically, the proposed fusion method achieved the smallest average lateral error of 5.2 cm, corresponding to reductions of 25.7%, 21.2%, and 24.6% compared with the LiDAR method, the vision method, and the semantic-segmentation method, respectively. In addition, it attained the highest navigation success rate of 95.4% and exhibited better stability in the RMS of the lateral error.

To assess stability from an operational-task perspective, 95% conservative bounds are reported for the key metrics in Table 5: a conservative upper bound for the lateral error and its RMS, and a conservative lower bound for the success rate. Under common approximations, these bounds are equivalent to worst-case robust estimates and can serve as surrogate evidence of long-term or large-scale operational stability [49]. As shown in Table 6 (converted from the values in Table 5), the proposed method yields a conservative upper bound of 6.18 cm for the lateral error, 7.78 cm for the RMS, and a conservative lower bound of 91.28% for the success rate—all superior to the two baseline methods. These results indicate that, even under a conservative regimen, the method maintains a lower error upper bound and a higher success-rate lower bound, demonstrating task-level stability for densely planted inter-row operation.

**Figure 18:**
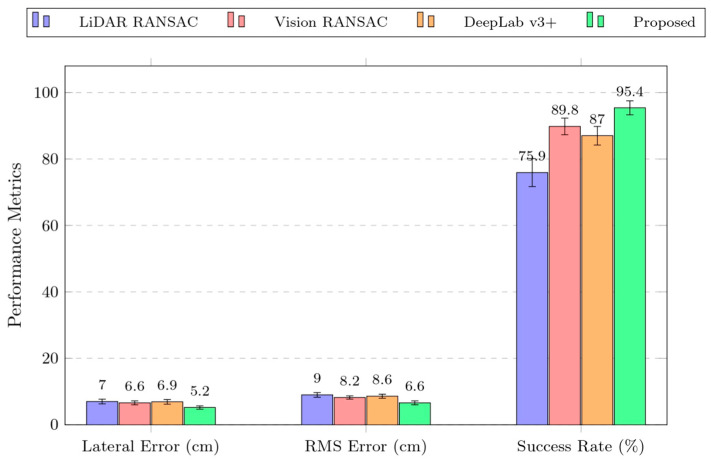
Comparison of different navigation line fusion methods.

As shown in Figure 18, a grouped bar chart provides an intuitive comparison of the navigation-line extraction performance of the different methods. The experimental results validate the effectiveness of the proposed vision and 2D LiDAR fusion-based approach in practical applications, offering a more feasible solution for agricultural automation.

## 4. Discussion

To improve the autonomous navigation capability of robots in densely planted pomegranate orchard environments, this study proposed a vision and 2D LiDAR fusion-based navigation line extraction method. The method integrates three core technical modules: YOLOv8-ResCBAM trunk detection, reverse ray projection data association, and geometric constraint navigation line fitting, achieving high-precision navigation line extraction in complex orchard environments.

In densely planted pomegranate orchards with challenging illumination and occlusions, our system achieves trunk detection with a current precision of 91.0%. To further improve performance, we will act on dataset collection/processing and model optimization: continuously add hard cases—strong backlighting, shadows, wet-ground glare, slight wind-induced sway, and season-dependent canopy density—apply targeted data augmentation and rigorous annotation to reduce small-object boundary noise; modestly improve image sharpness; jointly select, on the same validation set, suitable confidence thresholds and overlap-removal (NMS) thresholds to balance recall and precision; and apply simple temporal smoothing to detections across consecutive frames to avoid jitter from frame-wise flicker. These strategies are expected to raise the effective accuracy under complex outdoor conditions while maintaining stability across terrains and seasons, without noticeably increasing computational overhead.

The reverse ray projection algorithm proposed in this study demonstrates significant advantages in vision and LiDAR data association. Unlike traditional methods that project point clouds onto image planes, this algorithm projects visual detection results into three-dimensional rays and computes their intersections with LiDAR scanning planes, achieving more precise spatial correspondence. Experimental results show that in five groups of orchard environments with different densities (25–32 trees), the average association success rate reached 92.6% with a standard deviation of 0.9%, validating the algorithm’s stability and adaptability. The level of association success rate directly affects the accuracy of subsequent navigation line fitting. Association failures typically occur due to the following situations: first, sparse point clouds when LiDAR encounters small trunk diameters at long distances, leading to DBSCAN clustering failure; second, visual detection errors under complex lighting conditions, producing incorrect ray directions; third, irregular obstacles in orchards such as support poles and irrigation equipment interfering with data association.

To address the problem of traditional RANSAC algorithms being susceptible to noise in orchard environments, this study introduced a geometric constraint algorithm that guides the line fitting process by analyzing geometric relationships between fruit trees and constructing angle histograms. Experimental results show that compared to traditional RANSAC algorithms, the geometric constraint algorithm improved fitting accuracy by 2.4%, inlier ratio by 5.1%, and computational efficiency by 47.06%. The primary reason for performance improvement lies in the geometric constraint mechanism’s full utilization of prior knowledge about pomegranate orchards. Fruit tree rows in pomegranate orchards are typically planted along the same direction, and this regularity provides powerful constraints for the algorithm. By constructing direction histograms and extracting principal direction angles, the algorithm can preferentially select candidate lines that conform to orchard planting patterns during the fitting process, thereby improving real-time efficiency and stability of fitting.

In the orchard navigation-line tracking trials, the proposed fusion method delivered strong overall performance: the average lateral error was 5.2 cm, the RMS was 6.6 cm, and the navigation success rate reached 95.4%. Relative to the single-LiDAR method, the average lateral error and RMS decreased by 25.7% and 26.7%, respectively, while the success rate increased by 19.5%. Relative to the single detection-based vision method, the average lateral error and RMS decreased by 21.2% and 19.5%, with a 5.6% gain in success rate. Compared with the DeepLab v3+ semantic-segmentation method, the average lateral error and RMS decreased by 24.6% and 23.3%, and the success rate improved by 8.4%. For single LiDAR, when trunks are thin or the ground has soil cover or glare, near-ground returns become sparse and contain outliers, so the RANSAC-fitted centerline is prone to abrupt jumps. For single vision, strong backlighting and local occlusions easily cause misses/false detections and unstable localization of trees. The DeepLab v3+ method yields a smooth curve under sunny straight-row conditions, but under cross-slope, backlit, and occluded scenes its robustness is lower than the detection-based vision baseline; mask fragmentation and S-shaped skeletons are also common, making the fitting sensitive to noise. By using vision to provide semantic candidates that constrain geometric search, then weighting the fit with LiDAR’s absolute scale and directional consistency—and further applying a directional histogram and Kalman smoothing to suppress transient outliers—the proposed method markedly reduces line jumps and polyline jaggedness. Fusing the two sensors enables stable navigation across varying environmental conditions.

In densely planted inter-row scenarios, the method still exhibits a lower error upper bound and a higher success-rate lower bound under the conservative-bounds regimen, indicating task-level stability. The approach relies on three keys: a geometric prior of row-direction consistency in tree rows, the detectability of trunk-type targets, and the clusterability of sparse LiDAR returns. As shown by the derivation of the number of LiDAR hits on a trunk during navigation in Section 2.5.1 (Equation (8)), the number of points increases when trunks are thicker or the range is smaller (e.g., in apple or citrus orchards). Within reasonable parameter ranges across crops and tree forms, the return density is sufficient to support stable clustering and the subsequent geometrically constrained fitting, thereby providing clear parameter bounds and theoretical feasibility for cross-orchard transferability. To verify transferability, future work will add small-scale cross-scene tests—e.g., one test plot each in an apple and a citrus orchard—using few-shot fine-tuning only for the detection head while keeping the fusion and geometric-constraint fitting modules unchanged. We will also add task-level metrics (long-term navigation stability and obstacle-avoidance success rate) and report them alongside the average lateral error, RMS, and success rate.

Based on our data and field experience, we draw the following conclusions about environmental and meteorological impacts on measurement quality. Preferred conditions: clear days with stable, moderate illumination; no precipitation or fog; dry ground; ambient wind speed ≤ 3 m·s^−1^. Under these conditions, image illumination and shadow boundaries are most stable, ground-surface glare is minimal, spurious LiDAR returns are markedly reduced, and measurement quality is optimal. Adverse conditions: strong direct sunlight, higher wind speeds, rain, fog, or standing water. Strong direct sunlight produces long, sharp shadows and highlights that cause fluctuations in vision; LiDAR returns become mixed and outliers increase. Higher winds induce sway of branches and slender trunks, creating short-term occlusions and unstable tree localization. Rain/fog can fog the camera lens and LiDAR window, further degrading SNR. Recommended acquisition seasons and times: when compatible with production, prioritize concentrated data collection after pruning and before budbreak or in the early fruit-set stage (sparser canopy, less occlusion), and during dry or low-rainfall periods; avoid extended rainy or foggy periods whenever possible.

The results indicate that the proposed method can improve robot autonomous navigation capability in complex orchard environments and provide new insights for the popularization and promotion of orchard mechanized operations. In future work, the application will be extended to more types of orchard environments and different planting patterns to verify the algorithm’s generalizability and portability, aiming to provide effective technical solutions for robot navigation in densely planted orchard environments.

## 5. Conclusions

This study proposed a vision and LiDAR fusion-based inter-row navigation line extraction method for orchards, addressing the path extraction problem in densely planted pomegranate orchard environments. This research integrated a YOLOv8-ResCBAM trunk detection model, vision and LiDAR data fusion, and navigation line extraction algorithms under geometric constraints. In detection performance evaluation experiments, the YOLOv8-ResCBAM model improved trunk detection accuracy, achieving an mAP50 of 95.7% and a recall rate of 92.5%. The proposed reverse ray projection algorithm achieved precise data association with an association success rate of 92.6%. The proposed geometric constraint algorithm improved fitting accuracy by 2.4%, inlier ratio by 5.1%, and computational efficiency by 47.06% compared to traditional RANSAC algorithms. In the orchard navigation-line tracking trials, the vision and 2D LiDAR fusion-based navigation line extraction method reduced the average lateral error by 25.7%, 21.2%, and 24.6% relative to the single 2D LiDAR, single detection-based vision, and DeepLab v3+ semantic-segmentation methods, respectively, lowering the average lateral error to 5.2 cm and achieving a navigation-line extraction success rate of 95.4%. In addition, 95% conservative bounds were reported: a conservative upper bound of 6.18 cm for the lateral error, 7.78 cm for the lateral-error RMS, and a conservative lower bound of 91.28% for the success rate—all superior to the three baselines—demonstrating task-level stability for densely planted inter-row operation.

This study provides research concepts and solutions for robot autonomous navigation computation in densely planted orchard environments, offering reference value for advancing orchard mechanization and intelligent development. With the continuous improvement and optimization of related technologies, this method is expected to find broader applications in modern agriculture.

## Figures and Tables

**Table 1:** Hardware components.

Component	Parameter	Value
Intel RealSense D455	RGB Resolution	Up to 1280 × 800
	RGB Frame Rate/fps	Up to 90
	RGB FOV/(°)	86 × 57 (±3)
SLAMTEC 2D LiDAR	Measurement Radius/m	0.2–12
	Sampling Frequency/k	16
	Scanning Frequency/Hz	5–15
	Angular Resolution/(°)	0.225
	Scanning Range/(°)	360
	Ranging Accuracy	2% of actual distance (≤5 m)
NVIDIA Jetson XAVIER NX	CPU	6-core NVIDIA Carmel ARM v8.2 64-bit CPU
	GPU	384-core NVIDIA Volta™ GPU with 48 Tensor Cores
	Acceleration Unit	2 × NVIDIA (NVIDIA Deep Learning Accelerator)
	TOPS	21
	Memory	8 GB 128-bit LPDDR4
Tracked Mobile Platform	Dimensions/mm	750 × 550 × 850
	Motion Controller	STM32
	Number of Motors	2
	Rated Power/w	322
	Rated Speed/r·min^−1^	75

**Table 2:** Performance comparison of different detection methods in pomegranate trunk detection.

Detection Model	mAP50 (%)	Recall Rate (%)	Precision (%)	Inference Time (ms)
YOLOv8	95.5	91.6	91.4	12.96
YOLOv8-ECA	95.5	90.9	91.4	13.04
YOLOv8-GAM	95.2	91.1	91.6	15.64
YOLOv8-SA	95.6	91.6	92.0	13.71
YOLOv8-ResCBAM (Proposed)	95.7	92.5	91.0	15.36

**Table 3:** Data association statistics.

Test	Number of Fruit Trees	Average Association Count	Association Success Rate (%)
Test 1	25	23.0	92.1
Test 2	27	24.8	91.9
Test 3	30	27.7	92.3
Test 4	28	26.4	94.3
Test 5	32	29.6	92.5
Average	28.4	26.3	92.6

**Table 4:** Performance Comparison of GCA and RANSAC Algorithm.

Test Case	Tree Points	Algorithm	RMSE (m)	Inlier Ratio (%)	Runtime (s)
Test 1	19	GCA	0.1469	79.44	0.001
		RANSAC	0.1395	79.44	0.0087
Test 2	21	GCA	0.09	95.45	0.002
		RANSAC	0.1062	85.45	0.005
Test 3	27	GCA	0.0982	96.43	0.009
		RANSAC	0.0947	89.29	0.004
Test 4	23	GCA	0.0756	95.83	0.0017
		RANSAC	0.0794	91.29	0.005
Test 5	20	GCA	0.0775	95	0.0012
		RANSAC	0.0786	95	0.003
Test 6	20	GCA	0.1018	90	0.0013
		RANSAC	0.1059	85	0.005
Average	21.7	GCA	0.0983	92.03	0.0027
		RANSAC	0.1007	87.58	0.0051
Improvement	-	GCA vs. RANSAC	2.40%	5.10%	47.06%

**Table 5:** Performance comparison of different navigation line extraction methods.

Method	Average Lateral Error ± Standard Deviation/cm	Average Lateral Error RMS ± Standard Deviation/cm	Average Success Rate ± Standard Deviation (%)
LiDAR RANSAC	7.0 ± 0.7	9.0 ± 0.7	75.9 ± 4.2
Vision RANSAC	6.6 ± 0.6	8.2 ± 0.5	89.8 ± 2.5
DeepLab v3+	6.9 ± 0.7	8.6 ± 0.6	87.0 ± 2.8
Proposed Method	5.2 ± 0.5	6.6 ± 0.6	95.4 ± 2.1

**Table 6:** 95% Conservative Bounds at the Task Level.

Method	Conservative Upper Bound of Lateral Error/cm	Conservative Upper Bound of Lateral Error RMS/cm	Conservative Lower Bound of Success Rate (%)
LiDAR RANSAC	8.37	10.37	67.68
Vision RANSAC	7.78	9.18	84.90
DeepLab v3+	8.27	9.78	81.51
Proposed Method	6.18	7.78	91.28

Note: Calculation convention: conservative upper bound = mean + 1.96 × SD; conservative lower bound = mean − 1.96 × SD.

## Data Availability

The data presented in this study are available on request from the corresponding author. The data are not publicly available due to privacy and permissions re-strictions.
